# Effects of Anthocyanins on Vascular Health

**DOI:** 10.3390/biom11060811

**Published:** 2021-05-30

**Authors:** Ioana Mozos, Corina Flangea, Daliborca C. Vlad, Cristina Gug, Costin Mozos, Dana Stoian, Constantin T. Luca, Jarosław O. Horbańczuk, Olaf K. Horbańczuk, Atanas G. Atanasov

**Affiliations:** 1 Department of Functional Sciences-Pathophysiology, Center for Translational Research and Systems Medicine, “Victor Babes” University of Medicine and Pharmacy, 300173 Timisoara, Romania; 2 Department of Pharmacology and Biochemistry-Pharmacology, “Victor Babes” University of Medicine and Pharmacy, 300041 Timisoara, Romania; flangeacorina@yahoo.com (C.F.); dalivlad@yahoo.com (D.C.V.); 3 Timiş County Emergency Clinical Hospital, 300723 Timisoara, Romania; 4 Department of Microscopic Morphology-Genetics, “Victor Babeş” University of Medicine and Pharmacy, 300041 Timisoara, Romania; dr.cristina.gug@gmail.com; 5 Faculty of Medicine, “Victor Babes” University of Medicine and Pharmacy, 300042 Timisoara, Romania; mozoscostin@gmail.com; 6 2nd Department of Internal Medicine-Endocrinology, “Victor Babeş” University of Medicine and Pharmacy, 300723 Timisoara, Romania; stoian.dana@umft.ro; 7 Department of Cardiology–Cardiology II, “Victor Babeş” University of Medicine and Pharmacy, 300310 Timisoara, Romania; 8 Institute of Cardiovascular Diseases, 300310 Timisoara, Romania; 9 Institute of Genetics and Animal Biotechnology of the Polish Academy of Sciences, Jastrzebiec, 05-552 Magdalenka, Poland; olav@rocketmail.com; 10 Faculty of Human Nutrition, Warsaw University of Life Sciences, Nowoursynowska 159 C, 02-776 Warsaw, Poland; olaf_horbanczuk@sggw.edu.pl; 11 Ludwig Boltzmann Institute for Digital Health and Patient Safety, Medical University of Vienna, Spitalgasse 23, 1090 Vienna, Austria; 12 Department of Pharmacognosy, University of Vienna, Althanstraße 14, 1090 Vienna, Austria

**Keywords:** anthocyanins, arterial stiffness, endothelial function, cardiovascular risk, berries, antioxidants

## Abstract

Cardiovascular disorders are leading mortality causes worldwide, often with a latent evolution. Vascular health depends on endothelial function, arterial stiffness, and the presence of atherosclerotic plaques. Preventive medicine deserves special attention, focusing on modifiable cardiovascular risk factors, including diet. A diet rich in fruits and vegetables has well-known health benefits, especially due to its polyphenolic components. Anthocyanins, water-soluble flavonoid species, responsible for the red-blue color in plants and commonly found in berries, exert favorable effects on the endothelial function, oxidative stress, inhibit COX-1, and COX-2 enzymes, exert antiatherogenic, antihypertensive, antiglycation, antithrombotic, and anti-inflammatory activity, ameliorate dyslipidemia and arterial stiffness. The present review aims to give a current overview of the mechanisms involved in the vascular protective effect of anthocyanins from the human diet, considering epidemiological data, in vitro and in vivo preclinical research, clinical observational, retrospective, intervention and randomized studies, dietary and biomarker studies, and discussing preventive benefits of anthocyanins and future research directions.

## 1. Introduction

Cardiovascular disorders are leading mortality causes worldwide, often with a latent evolution. Vascular health depends on endothelial function, arterial stiffness, and the presence of atherosclerotic plaques, and it predicts, if impaired, future major cardiovascular events [1,2,3,4]. Preventive medicine deserves special attention, focusing on modifiable cardiovascular risk factors, including diet. The type and amount of food can influence other cardiovascular controllable risk factors: serum cholesterol, blood pressure, diabetes mellitus, and obesity. A diet rich in fruits and vegetables has well-known health benefits, especially due to its polyphenolic components [5,6,7,8,9,10,11]. 2021 is the International Year of Fruits and Vegetables, according to the General Assembly of the United Nations, which emphasizes the importance of the present topic. The biological and pharmacological effects of dietary natural products have been intensively studied, and the research might have been also fueled by the growing industry related to natural products utilization [12].

**Figure 1:**
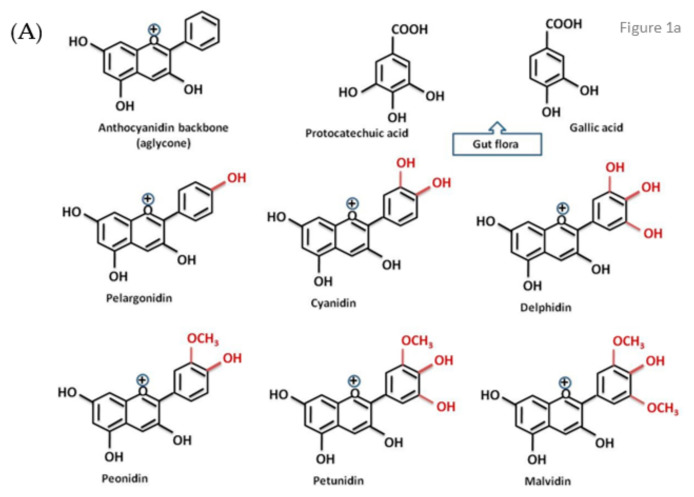

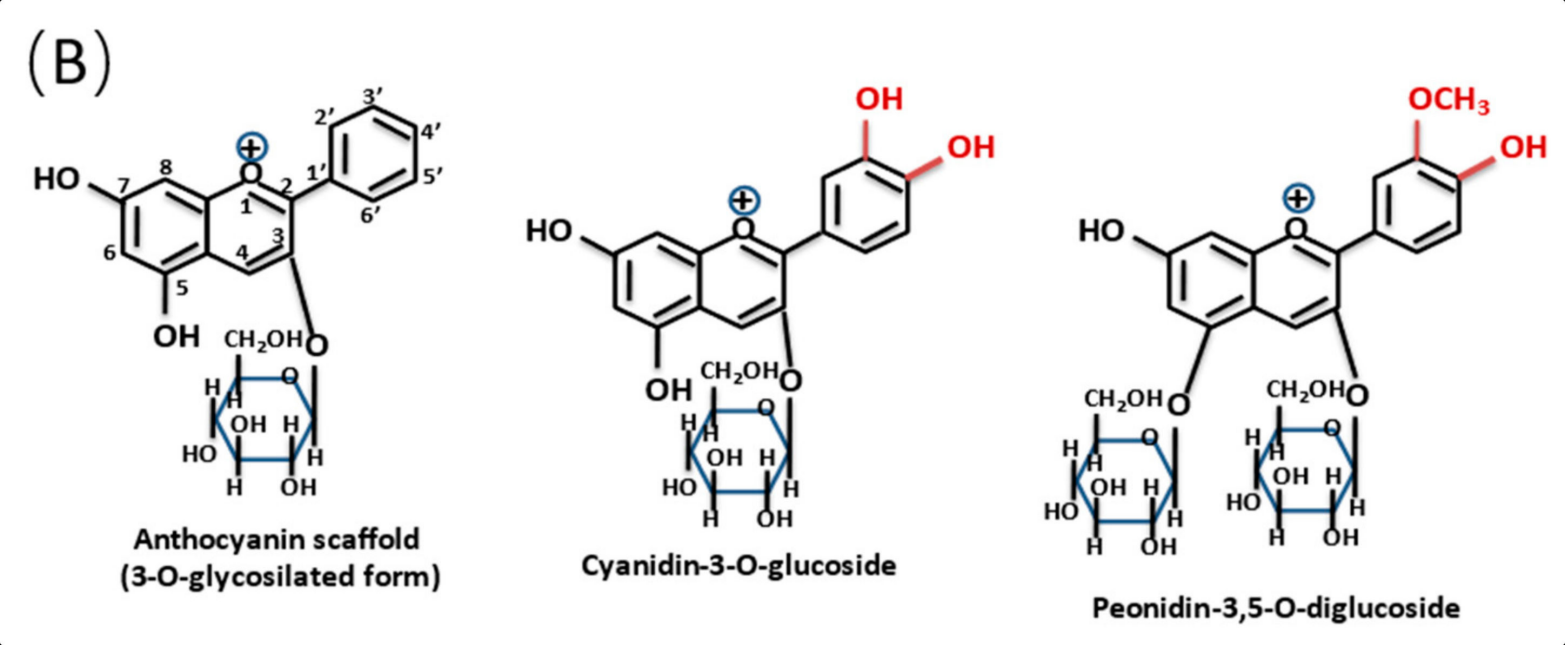
Chemical structures of the most common anthocyanidins (**A**) and their metabolites, and anthocyanins (**B**).

Anthocyanins (ACYs), water-soluble flavonoids, are responsible for the orange or red-blue color in flowers, seeds, fruits, and vegetables [13]. Chemically, anthocyanins include the sugar-free *anthocyanidine* aglycons and the anthocyanin glycosides [13] (Figure 1). The glycosylated structures are known as anthocyanins, while the non-glycosylated are the anthocyanidins, the precursors of anthocyanins [14]. The most common glycosylation site is position 3 where glucose is found usually as single sugar, especially for cyanidin. In some cases, an additional glucose may be attached in position 5, more often seen for peonidin, pelargonidin and delphinidin. Other sugars bound in position 3 were described in some studies, such as galactose and arabinose, as well as complex oligosaccharides such as rutinose, sophorose and sambubiose [14,15]. They are commonly found in berries, especially strawberries and blueberries, red grapes, apples and pears, blackcurrants, chokeberry, plums, cherries, nectarines, peaches, pomegranate, avocados, bananas, dates, nuts (almonds, hazelnuts, pistachio, pecan nuts), black rice, purple corn, cauliflower, red radishes, beans, cabbage, beets and onions, red and black carrots, purple sweet potato, beans, pepper, eggplant, black olives, and red lettuce, and fruit-derived products like red wine, juices, jam, and marmalade (Table 1) [8,16,17,18,19,20,21,22,23,24,25,26]. During berry ripening, anthocyanin content rises and is responsible for its health benefits [27]. Many anthocyanin-rich fruits are included in the Mediterranean diet and lifestyle, with favorable health effects. ACYs may also represent a source of natural food colorants [28].

Ingested ACYs undergo oral transformation due to salivary amylase. In the stomach ionic forms of ACYs were identified, which are hydrolyzed by several enzymes in the small intestine resulting in conjugated products, or simpler phenolic compounds, which are hardly absorbed and processed by the gut microbiota as free anthocyanidins and protocatechuic acid [27,29,30]. ACYs are the least well-absorbed polyphenols [31]. It has been reported that more anthocyanin can be absorbed with increasing dose [32]. Some biological activities of ACYs are due to the synergetic effect of their colonic catabolites [30]. Low-molecular-weight catabolites produced by the large intestine can be excreted in the feces within 2–8 h or absorbed again [30]. ACYs undergo dehydroxylation by colonic bacteria, to form hydroxybenzoic acid, followed by conjugation with glycine to form hippuric acid [33]. Anthocyanins and their catabolites also undergo phase 2 enzymatic metabolism, generating the glucuronidated, sulphated, and methylated forms, which persist in the urine for a long time after their intake, related, probably, to their transport in the bile [27]. On the other hand, colonic fermentation of ACYs increases beneficial bacteria, such as Bifidobacterium, Actinobacteria, Bacteroidetes and Lactobacillus [30,34]. The daily intake of anthocyanins can be estimated according to food databases [30]. Single doses of 150 mg to 2 g total anthocyanins given to volunteers, generally in the form of berries, resulted in extremely low plasma concentrations (10–50 nmol/l), and the maximal concentration was reached after 1.5 h in the plasma [31]. The most important factors related to the level of anthocyanins and their metabolites in our organism include: the ability to cross membranes, pH, enzymes of the digestive tract, microbiota, biliary acids, and food matrix [30].

The most important ACYs are, as follows: delphinidin, cyanidin, malvidin, pelargonidin, peonidin, and petunidin (Figure 1a) [13]. Several beneficial effects of ACYs were attributed to protocatechuic acid, a bioactive compound, synthesized by the gut microbiota from ACYs [35,36]. ACYs conjugated with sugar residues might be accompanied by anthocyanidins, the sugar-free parts of ACYs, with lower stability due to missing sugars [37]. One-half cup of blueberries provides about 121 mg of ACYs and consuming 1–2 portions daily can reduce cardiovascular risk [19,27]. Ponzo et al. also demonstrated the relationship between high ACYs intake and low incidence of cardiovascular events and all-cause mortality [38].

Considering that many people prefer natural therapies, the present paper aims to give a current overview of the vascular protective mechanisms attributed to anthocyanins in the diet, considering epidemiological data, in vitro and in vivo preclinical research, clinical observational, retrospective, intervention, and randomized studies, dietary and biomarker studies, and discussing preventive benefits of anthocyanins and future research directions.

**Table 1:** Anthocyanin content of the fruits and vegetables mentioned in the manuscript.

Fruits and Vegetables	Anthocyanin Content	Administrated as	References
Blackberry (*Rubus fruticosus)*	820–1800 mg/kg	Fresh fruit	[39]
Black mulberry (*Morus nigra*)	42.4 mg/100 g	Fresh fruit	[40]
Bilberry (*Vaccinium myrtillus*)	1610–5963 mg/L	Juice 100%	[23]
Black carrots (*Daucus carota* ssp. *sativus* var. *atrorubens*)	1750 mg/kg	Fresh vegetable	[41]
Black chokeberries (*Aronia melanocarpa*)	1480 mg/100 g	Fresh fruit	[42]
Black soybean (*Glycine max*)	0.1–23.04 mg/g	Seed coat	[43]
Black currant (*Ribes nigrum*)	176–1298 mg/L	Juice 100%	[23]
Blood orange (*Citrus sinensis*)	4.6 ± 0.7; 72.4 ± 0.6 mg/L	Fresh fruit	[44]
Blueberry (*Vaccinium virgatum* and *Vaccinium corymbosum*)	134 mg/kg	Fresh fruit	[45]
Cherry (*Prunus cerasus*)	22 mg/100 g	Fresh fruit	[23]
Cornelian cherry (*Cornus mas*)	128.45 ± 5.14 mg/L C3G 226.78 ± 8.61 mg/L		[46]
Cowpea (*Vigna unguiculata*)	1.7–3.9 mg/g	Seeds	[43]
Cranberry (*Vaccinium macrocarpon*)	460–2000 mg/kg	Fresh fruit	[39]
Eggplant (*Solanum melongena* L.)	11.53 g/100 g DW delphinidin, 0.55 g/100 g DW of petunidin	Fruit	[25]
Grape (*Vitis vinifera*)	300–7500 mg/kg	Fresh fruit	[39]
Kiwi (*Actinidia melanandra*)	478 μg/g in skin, 81 μg/g in flesh	Fresh fruit	[47]
Mahaleb cherries (*Prunus mahaleb*) (g/kg DW)	7.80 ± 1.10; 15.60 ± 3.10; 17.70 ± 3.50; 18.90 ± 0.90	Fresh fruit	[48]
Pepper (*Capsicum annuum* L.)	0.96 mg anthocyanin/100 g fresh weight		[26]
Pomegranate (*Punica granatum*)	43 mg/L	Juice	[23]
Purple maize (*Zea mays indurate*)	4.3 to 117-mg C3G/g	dark-colored purple corncob	[49]
Purple sweet potato (*Ipomoea batatas* L.)	0.94- 1.75 g/kg	Fresh weight	[24]
Strawberry (*Fragaria × ananassa*)	232 mg/100 g	Fresh fruit	[23]

C3G = cyanidin 3-glucoside; P3OG = pelargonidin-3-O-glucoside; DW = dry weight.

## 2. Preclinical and Clinical Research and Molecular Mechanisms

### 2.1. Metabolic Effects of Anthocyanins

#### 2.1.1. Clinical Research

Acylated anthocyanins of black carrots *(Daucus carota* ssp. *sativus* var. *atrorubens)* have an anti-oxidative effect (cyanidin-3-O-glucoside), can improve plasma lipid profile, by lowering the LDL-cholesterol levels and serum triglycerides and increasing the HDL-cholesterol levels, and may improve glucose tolerance and insulin resistance (dephinidin-3-O-rutinoside) [50,51,52,53,54]. Dohadwala et al. reported a modest reduction of HDL-cholesterol after 4 weeks of cranberry *(Vaccinium macrocarpon)* juice consumption in patients with coronary heart disease, related, probably, to the characteristics of the study population and short treatment period, because Ruel et al. revealed an increase of HDL-cholesterol in obese patients after 12 weeks supplementation with cranberry juice [55,56]. Nonesterified fatty acids also decreased after ACYs supplementation in a rat model of metabolic syndrome [36].

The main mechanisms explaining the reduction of total cholesterol levels include increasing fecal excretion of sterols, down-regulation of the gene expression of the hepatic HMG-CoA reductase, reduction in serum apo B- and apo-CIII-containing triglyceride-rich particles, inhibition of cholesteryl-ester transfer protein, slowing intestinal lipid absorption and increase of the expression of LDL-receptor [51,53]. Dietary fibers, the main components of fruits and vegetables, can also reduce LDL and short-chain fatty acids, as well as liver cholesterol synthesis [52].

*Strawberry (Fragaria × ananassa)* supplementation in healthy volunteers, 500 g daily for 1 month, reduced total and LDL cholesterol and triglycerides levels, without any influence on HDL values [57]. Placebo-controlled studies demonstrated an increase of HDL- cholesterol level and functionality in patients with dyslipidemia after ACYs supplementation [58].

Anthocyanin intake had a stronger association with weight control, enabling weight maintenance, in a study including more than 120,000 patients, followed for up to 24 years, included in the Health Professionals Follow-up Study, Nurses’ Health Study, and Nurses’ Health Study II, compared to flavonols, flavan-3-ols and flavonoid polymers [59]. Preventing even small amounts of weight gain can reduce cardiovascular risk [59]. ACY intake was associated not only with a lower fat mass, but may also influence central adiposity, independent of genetic and environmental factors [20].

#### 2.1.2. Preclinical Research

ACYs from cornelian cherry *(Cornus mas)* increased expression of peroxisome proliferator-activated receptor (PPAR) alpha and gamma in the liver, contributing to the antiatherosclerotic effect [7]. Inhibition of PPARγ2 expression, an adipogenic transcription factor, impairs hepatic lipid accumulation and lipogenesis as effects of black chokeberries *(Aronia melanocarpa)*, known to be rich in ACYs [60]. Black chokeberries prevent lipotoxicity and hepatocellular injury, delaying the progression of nonalcoholic fatty liver disease (NAFL) to nonalcoholic steatohepatitis (NASH) and liver cirrhosis [60]. ACYs down- regulate key enzymes required for cholesterol and fatty acid synthesis and activates free fatty oxidation [24]. Cyanidin-3-O-glucoside was reported to up-regulate the lipase gene and enhance the lipolytic activity of rat adipocytes [61].

Several animal studies demonstrated the benefits of ACYs on reverse-cholesterol transport and HDL formation, via regulation of lipids transporters and increase of paraoxonase 1 activity [58]. Anthocyanidin-3-glucoside promotes reverse cholesterol transport mediated by its gut microbiota metabolite, protocatechuic acid [29]. ACYs from *purple maize (Zea mays indurate)* and chokeberries were able to reverse or attenuate metabolic syndrome, due to their anti-inflammatory effect, preventing inflammatory cell infiltration into the tissues, in male Wistar rats [36].

ACYs also improve the carbohydrate metabolism, impairing intestinal absorption of glucose by inhibiting alpha-glucosidase and alpha-amylase, protecting pancreatic beta-cells from oxidative stress, and normalizing cardiac NADPH oxidase expression [53,62,63].

Anthocyanins reduce the expression of neuropeptide Y, modulating appetite and food intake, and increase gamma-aminobutyric acid receptor, reducing protein kinase A-alpha and phosphorylated cAMP-response element-binding protein in the hypothalamus, controlling body weight and adipose tissue size [50,64]. Additionally, fibers that often co-occur with ACYs in food can decrease postprandial glucose by reducing gastric emptying times [28]. Pelargonidin 3-glucoside-enriched strawberries reduced abdominal fat and body weight gain in rats with metabolic syndrome induced by a diet rich in carbohydrates and fats [65]. Obesity includes, besides excess adipose tissue, also macrophages infiltration, and inflammation, which lead to insulin-resistance [66]. The anti-inflammatory effect of ACY and the effect on insulin sensitivity also enable weight loss. The anti-obesity effects of ACYs are also related to changes in adipocytokine expression (up-regulation of adiponectin and down-regulation of plasminogen activator inhibitor-1 and interleukin-6), and upregulation of gene expression in adipocytes [61,67].

Concluding, anthocyanins exert hypolipidemic and anti-obesity effects, also improving glucose metabolism and insulin sensitivity (Table 2).

### 2.2. Effects of Anthocyanins on Endothelial Function

Endothelial dysfunction is a key mechanism in the development of atherosclerotic plaque, related to the loss of the barrier function, proinflammatory and prothrombotic effects, and reduction of nitric oxide (NO) [72]. Many cardiovascular risk factors can cause endothelial dysfunction, including hypertension, smoking, dyslipidemia, diabetes mellitus [72]. Endothelial dysfunction is a hallmark of several cardiovascular disorders, including hypertension and coronary heart disease [87]. The endothelial function may be assessed using flow-mediated vasodilation, on the brachial artery, or ultrasonography, defined as a change in brachial artery diameter in response to hyperemia [88,89].

Anthocyanin-rich foods and beverages can activate endothelial NO synthase and improve endothelial function in vitro and in vivo [71,72] (Table 2). Gallic acid, a microbiota anthocyanin metabolite, can increase NO levels by increasing phosphorylation of endothelial NO synthase [29]. The vasodilator effect of NO is related to its action on the smooth muscle cells and production of cGMP [72]. Considering that the half-life of NO is short, cGMP can be used as an index of NO activity and endothelial function [72]. ACYs protect the endothelial cells due to the activation of the nuclear factor 2 pathway (Nrf2), which regulates the NO synthase and production of NO [75]. Daily blueberry (*Vaccinium virgatum* and *Vaccinium corymbosum)* consumption reduced blood pressure and arterial stiffness, related probably to increase of NO production [90]. NO synthase inhibitors abolish the effect of ACYs on endothelium-dependent vasodilation in human subjects and rats [72]. Tabart et al. demonstrated that amplitude of vascular relaxation after blackcurrant (*Ribes nigrum*) juice supplementation on isolated porcine coronary artery rings was correlated to the total ACYs content and concentration and not their antioxidant capacity [91]. Anthocyianidins also improve endothelial function by impairing the expression of endothelin-1 [73]. Estradiol and ACYs with phytoestrogenic properties activate NO synthase via interaction with estrogen receptors [92]. Blackcurrant anthocyanins increased endothelial NO synthase mRNA expression and NO production in human endothelial cells and an ovariectomized rat model [92].

The endothelial protective effects of ACYs are also related to their antioxidant activity [74]. Luteolinidin is an *anthocyanidin* that can improve endothelial function due to its antioxidant properties [86].

The low plasma concentration and oral bioavailability of flavonoids question their involvement in improving endothelial function, but there is a membrane transporter bilitranslocase, a bilirubin-specific transporter, able to rapidly mediate the uptake of several flavonoids, including cyanidin-3 glucoside into the endothelial cells [74]. Moreover, Bharat et al. indicated that the vascular benefits of blueberry ACYs are due to their metabolites, hippuric, hydroxyhippuric, benzoic, vanillic, and isovanillic acid in a study on human aortic endothelial cells [93]. However, intact ACYs are also responsible for the improvement of flow-mediated vasodilation [72]. Vascular benefits include improvement of endothelial function and NO production, anti-inflammatory and antioxidant effect [93].

It was suggested that ACYs can be incorporated into the membrane and into the cytosol of the endothelial cells, preventing the occurrence of endothelial dysfunction and protecting against oxidative stressors [57,70]. Increased levels of nonesterified fatty acids (NEFAs) inhibited aortic endothelial nitric oxide synthase, causing hypertension [36]. ACYs can reduce NEFAs, thus improving endothelial NO synthesis [36].

Thandapilly et al. demonstrated an improved arterial relaxation and a significant reduction in blood pressure and attenuated cardiac hypertrophy in spontaneously hypertensive rats after freeze-dried *grape (Vitis vinifera) powder* administrated for 10 weeks [94]. Red grapes contain a variety of polyphenols, including ACYs [94].

Findings, whether the effects of ACYs are just acute or also chronic on endothelial function, are conflicting. *Cranberry* juice, a mix of polyphenols, especially ACYs, showed just an acute and no chronic benefit on flow-mediated vasodilation (FMD) of the brachial artery, in patients with coronary heart disease, probably due to severely impaired endothelial function in those patients, but improved arterial stiffness [56]. Several other clinical studies reported just an acute and no chronic benefit on endothelial function, related to the kinetics of ACYs [56,95]. Most of the chronic intervention studies reported improvements in vascular function, especially FMD [96]. It was suggested that ACYs may influence the composition of the arterial wall, and the effect could persist for a longer time, but additional studies are required to confirm such mechanism [56]. Anyway, such effects would represent no surprise, since ACYs have been shown to increase skin levels of collagen and elastin in ovariectomized rats [97]. Anthocyanin plasma metabolites from blueberries caused both acute and chronic flow-mediated dilation improvements in mice [98].

### 2.3. Anti-Inflammatory Effects of Anthocyanins

Dietary ACYs have been shown to reduce systemic and vascular inflammation in several studies [99]. Atherosclerosis is a chronic inflammatory disease, and the anti-inflammatory effect of ACYs can slow down the atherosclerotic process [99]. ACYs were already mentioned to reverse or attenuate metabolic syndrome in rats [36]. Anthocyanins exert their anti-inflammatory effects by activating the nuclear factor 2 pathway (Nrf2), impairing overproduction of inflammatory cytokines in response to oxidative stress and of chemokines in response to inflammation, also limiting NF-k beta activation and inhibiting the expression of vascular smooth muscle cell adhesion molecule and COX-2 expression in vascular smooth muscle cells [75]. Suppression of anti-arthritic effects of black soybean (Glycine max) seed coats was also mediated via suppression of NF-k beta signaling [100]. ACYs can suppress the adhesion of monocytes to the endothelium [101,102,103]. The anti-inflammatory and antioxidant effects of ACYs depend on their structure. Non-acylated ACYs, from mahaleb cherries (Prunus mahaleb) or blackcurrant, had higher anti-inflammatory and antioxidant activity compared to ACYs acylated with cinnamic acid (black carrot and “Sun Black” tomato) [103]. The explanation is related to a more important inhibitory effect of non-acylated anthocyanins on TNF-α-induced expression of adhesion molecules in endothelial cells than acylated anthocyanins [103]. Berries with higher cyanidin content, especially blackberries (Rubus), chokeberries, and bilberries (Vaccinium myrtillus), are more likely related to the anti-inflammatory effect [18].

Anthocyanins significantly decreased the levels of inflammatory markers such as high sensitivity C-reactive protein, soluble vascular cell adhesion molecule-1, and plasma interleukin-1β in hypercholesterolemic patients [99]. ACYs supplementation for 4 weeks also decreased high sensitivity C-reactive protein levels in patients with metabolic syndrome [104]. In cell culture assays, an anthocyanin mixture inhibited interleukin 6 and C reactive protein production and vascular cell adhesion molecule 1 (VCAM-1) secretion, in a dose-dependent manner [99]. The anti-inflammatory effect of the ACYs mixture was stronger when compared with the effects of delphinidin and cyanidin separately, and different anthocyanin compounds had additive or synergistic effects in mediating the anti-inflammatory activity [99]. The modulation of vascular inflammation due to ACYs is related to gene expression changes, affecting cell adhesion, migration, immune response, and cell differentiation [98]. On the other hand, fibrinogen increased after two months with black chokeberry extract in patients with metabolic syndrome, despite benefits for blood pressure, serum level of endothelin-1, lipids, and oxidative status [105].

Concluding, ACYs, especially non-acylated ACYs, modulate vascular inflammation, preventing over-infiltration with immune cells, which is a vessel protecting mechanism, exerting also anti-aging effects. The anti-inflammatory effects of ACYs were revealed monitoring C reactive protein and high sensitivity C-reactive protein level, as well as adhesion molecules. Data are missing related to other inflammatory markers, associated with atherosclerosis and plaque instability, such as serum amyloid A, pentraxin, apolipoprotein-associated phospholipase A2, and soluble CD40 ligand [106]. ACYs deserve special attention when assessing the inflammatory potential of the diet [107].

### 2.4. Anthocyanins as Antioxidants

Oxidative stress is involved in the pathophysiology of arterial stiffness, impairs endothelial function due to uncoupling of endothelial NO synthase, and damages endothelial proteins, lipids, and DNA [3]. Reactive oxygen species (ROS) cause oxidative changes of tetrahydrobiopterin and cysteins, resulting in superoxide and not NO production [87,108].

ACYs have higher antioxidant activity compared to other flavonoids. Their ability to limit oxidative stress has been extensively studied in vitro and in vivo [13,37,78,81]. Due to the fast metabolism of ACYs, their maximal plasmatic antioxidant value is reached between 15 and 30 min after ingestion [53]. In vivo studies revealed higher radical scavenging activity and decreased free-radical production due to ACYs [37,82].

As already mentioned, anthocyanins protect pancreatic beta-cells from oxidative stress induced by glucose, reduce lipid peroxidation, and the negative effects of ROS [62,75]. Activation of the nuclear factor Nrf2 pathway regulates the expression of antioxidant proteins, able to protect against injury and inflammation [75]. The antioxidant effect was also attributed to protocatechuic acid, a metabolite of ACYs, and was also demonstrated in deoxycorticosterone acetate-salt hypertensive rats [35,36,109]. Luteolinidin, a potent antioxidant, and radical scavenger inhibits CD38, increasing the myocardial and endothelial NAD(P) pool and facilitating NO production [87]. Ischemia/reperfusion of the heart causes CD38 activation, and luteolinidin, as a CD38 inhibitor, preserves cardiac function and reduces myocardial infarction size after reperfusion [87].

Several clinical studies confirmed the antioxidant effect of ACYs. Strawberry supplementation, in 23 healthy volunteers, resulted, after 1 month, in a decrease of oxidative stress biomarkers such as serum malondialdehyde, urinary 8-OHdG, and isoprostanes, and increased total plasma antioxidant capacity [57]. Lynn et al. found an increased antioxidant status in healthy adults, measured as the ferric reducing ability of plasma, after intake of cherry (Prunus cerasus) juice concentrate, rich in ACYs [17].

Cyanidin-3-O-glucoside, the most widely distributed anthocyanin, is a good scavenger of superoxide, but not hydroxyl radicals, with a pH-dependent oxidative potential [53]. Cyanidin, from grape seeds extract, protects DNA against oxidation, better than catechin [83]. Despite the antioxidant effect, activation of the Nrf2 increases cholesterol in the plasma and liver, promoting the atherosclerotic process [110].

The antioxidant effects of anthocyanins contribute to the improvement of endothelial function and arterial elasticity. Antioxidant effects were reported especially for delphinidins, protocatechuic acid and luteolinidin, and they all should be considered when assessing dietary antioxidant properties.

### 2.5. Other Vascular Effects of Anthocyanins

Nrf2 activated by ACYs exerts several anti-atherosclerotic effects, inhibiting the proliferation of vascular smooth muscle cells, reducing the level of oxidized LDL by activating CD 36 scavenger receptor [75]. Jiang et al. reported a reduced intima-media thickness and reduced atherosclerotic injuries, besides an improved lipid profile and atherogenic index, as well as decreased malondialdehyde content and increased anti-oxidative activity in atherosclerotic rats after black mulberry *(Morus nigra)* extract, rich in ACYs [40]. Extracts from boiled cowpea, a widely produced pulse grain, as well as whole seeds of cowpea, containing high amounts of anthocyanidins, inhibited LDL oxidation in humans [28]. The beneficial effect of some ACYs on atherosclerosis is mediated by gut microbiota metabolites, considering that ingested dietary ACYs are partly absorbed, while large amounts enter the colon and are degraded by gut microbiota as free anthocyanidins and protocatechuic acid [29].

Normal platelet function is important for cardiovascular health [85]. ACYs improve platelet function, impairing platelet aggregability [73,85]. Strawberries, rich in ACYs, significantly reduced the number of activated platelets in healthy controls, after 1 month of supplementation [57]. ACYs supplements also significantly decreased ADP-induced platelet activation in patients with metabolic syndrome [104]. In other words, ACYs prevent thrombosis.

Inhibition of angiotensin-converting enzyme (ACE) activity by delphinidin- and cyanidin-3-O-sambubiosides from *Hibiscus sabdariffa*, widely used in Mexico, explains probably the antihypertensive effect of the mentioned ACYs [86]. The antihypertensive effect was also attributed to protocatechuic acid in deoxycorticosterone acetate-salt hypertensive rats [109] and was confirmed by several clinical studies [111]. ACYs have been shown to prevent not just hypertension, but also cardiac hypertrophy [62]. Gallic acid, a microbiota anthocyanin metabolite, inhibited ACE, reducing blood pressure in spontaneously hypertensive rats (SHR), an effect comparable to captopril [29].

ACYs are also effective in metabolic syndrome by controlling serum lipids, glucose tolerance, insulin resistance, and blood pressure [36,52]. ACYs interact with the microbiota, acting as a prebiotic agent, decreasing the access of bacterial components into the body, and producing several metabolites able to reduce systemic inflammation and impair lipid uptake by adipocytes and release of adipokines [112].

Nanashima et al. compared the effects of an anthocyanin-rich blackcurrant extract and 4 blackcurrant ACYs, revealing phytoestrogenic activity of ACYs, related to the regulation of the metabolism of extracellular matrix components in the skin [97]. Further studies should provide insights related to the influence on the composition of the arterial wall.

### 2.6. Anthocyanins and Gene Expression

Genetic effects are involved in the regulation of ACYs biosynthesis in several fruits, such as *grapes* and red-fleshed kiwi *(Actinidia melanandra)* and vegetables [13,113,114]. In human subjects, besides down-regulation of the gene expression of the hepatic HMG-CoA reductase, which was already mentioned, the cardiovascular effect of ACYs is related to their gene expression modulating effect [115]. Supplementation with a bilberry anthocyanin-rich extract attenuated atherosclerotic injuries in apolipoprotein E-deficient mice [115]. The explanation relies on impairing mRNA levels of genes related to atherosclerosis in cultured macrophages and endothelial cells [115]. The nutrigenomic analysis identified 1261 genes modulated by the ACY-rich extract in the aorta, including down-regulation of genes involved in oxidative stress, coding for adhesion molecules or angiogenesis, and the up-regulation of genes associated with increased cell adhesion and decreased paracellular permeability [115]. Wild blueberry (*Cyanococcus*) consumption influenced the expression of more than 600 genes and 3 microRNAs, related also to an increase in peripheral blood mononuclear cells [98].

Genetic influences of ACYs were also mentioned related to their anti-inflammatory effect, reducing pro-inflammatory genes (NFKB1, PTGS2) [53]. ACYs upregulate also gene expression in adipocytes, especially lipid metabolism and signal-transduction genes [61].

## 3. Population-Based Studies

### 3.1. Observational and Intervention Studies

A few observational and intervention studies focused on the vascular effects of ACYs, revealing a reduction of arterial stiffness [116], endothelial function, and serum lipids [117,118] (Table 3). ACYs were administrated as chokeberry or pomegranate *(Punica granatum)* juice [117,118].

### 3.2. Randomized Controlled Trials (RCT)

Related to the acute arterial effects of ACYs, they can increase exercise performance. Blackcurrant extract increased femoral artery diameter during a submaximal sustained isometric contraction of the quadriceps muscle, emphasizing the ergogenic effects of this polyphenols [21]. Vasodilation of the femoral artery was accompanied by a hemodynamic response with a decrease of systolic, diastolic, mean arterial pressure and peripheral vascular resistance, increased cardiac output and stroke volume, and increased hemoglobin content in the vastus medialis [21].

Matsumoto et al. used near-infrared spectroscopy (NIRS) in 9 healthy men to measure left forearm blood flow (FBF) after venous occlusion and muscle oxygen consumption after arterial occlusion, before and hourly, for 4 h, after ingestion of blackcurrant anthocyanin [119]. Left forearm blood flow increased for 2–5 h after ACYs administration, with no significant difference in muscle oxygen consumption between ACYs and placebo intake [119]. The same article demonstrated increased peripheral blood flow and reduced muscle fatigue, improving shoulder stiffness after typing for 30 min if ACYs were previously administrated, but no improvement in typing performance [119].

Zhu et al. combined a short-term crossover study and a long-term interventional trial (12 weeks) in patients with hypercholesterolemia [72]. The maximal plasma concentration of delphinidins and cyanidins was obtained 1 h after dietary ACYs and was associated with the highest flow-mediated dilation (FMD) and plasma cGMP [72]. Long-term ACYs supplementation significantly increased FMD, cGMP, and HDL-cholesterol, and decreased vascular adhesion molecule-1 and LDL cholesterol [72]. Endothelial dysfunction in patients with hypercholesterolemia is related to dyslipidemia and inflammation, which were ameliorated by ACYs [72]. Zhu et al. also reported positive correlations between the changes in cGMP and HDL cholesterol concentrations and FMD in the ACYs group, and the disappearance of the endothelial effects of ACYs in the presence of NO-cGMP inhibitors [72].

FMD was also improved in a study including overweight or obese healthy participants of European origin, after 2 weeks of blood orange juice intake [120]. Blood pressure, lipid profile, high-sensitivity C-reactive protein, and endothelin-1 were not affected by the intervention. The authors concluded that the endothelial function was improved, and NO bioavailability increased due to the combined actions of ACYs and flavanone metabolites [120].

The longest RCT, including 115 patients with metabolic syndrome, revealed that 150 g blueberries/day for 6 months, resulted in improvement of endothelial function, arterial stiffness, and lipid profile, with no effect on insulin sensitivity [121]. There is a synergy between endothelial dysfunction and arterial stiffness, which might explain the vascular benefits of ACYs [121].

Smoking impairs endothelial function [122]. Blackcurrants administrated before smoking can attenuate the decrease in FMD in young smokers [123]. In other words, inadequate dietary intake of ACYs can contribute to cardiovascular disease in smokers, related, probably, to oxidative stress, which impairs nitric oxide production [122,123]. Adding vitamin E to ACYs increased endothelial functions in healthy nonsmokers as well as smokers [123]. Not all studies revealed the antioxidant effects of ACYs as a benefit for the endothelial function [124]. Balestra et al., revealed an independent antioxidant effect of ACYs, probably involving cellular signaling modulation [124].

The combination of ACYs with bromelain, a protein-digesting enzyme derived from pineapples, is beneficial for vascular health in humans, improving endothelial function, BP, antioxidant effect and oxygen utility capacity [125].

The majority of RCTs included in this review reported significant improvements in arterial stiffness following acute and chronic consumption of anthocyanin-rich foods, in patients with diverse disorders, body mass index, and age (Table 4).

## 4. Study Limitations

Contradictory results may be explained by low bioavailability, variable concentrations and doses, instability and different pharmakokinetics depending on the source, destabilization and storage of anthocyanins, different experimental setups, methodologies, follow-up periods, differences between in vivo and in vitro studies, concomitant medication which may influence vascular function or other polyphenol-containing foods and beverages throughout the study period.

The concentration of ACYs varies among cultivars [52]. Methods of processing and their duration may also influence ACYs content and effects, certain components are more affected than others by cooking [22,53]. As an example, boiled cowpea caused a more pronounced decrease of glycemic index than fried or mashed cowpea [132]. Heat, light, pH, structure, oxygen, solvents, metal ions, enzymes, other flavonoids, proteins, co-pigments, and storage may destabilize and degrade ACYs [52,53,96]. On the other hand, high light intensity, blue and red light and UV-A irradiation stimulate ACYs production in several plants due to the influence on biosynthetic genes [13]. Low temperatures also induced ACYs accumulation in several vegetables [13]. Heating enables anthocyanin degradation, but its stability depends on the source of the flavonoid. ACYs from black carrot have higher heat stability compared to those from sour cherry, grape or citrus juices, elderberry, and strawberry [52]. Gerardi et al. reported preservation of the phenolic content of tomato puree enriched with several anthocyanin-rich food colorants after pasteurization, as well as a higher antioxidant capacity [133]. ACYs from black carrot and blood orange include acylated anthocyanins, with a higher stability in neutral or acidic media [52]. Results also showed a significant decrease in anthocyanin stability at pH above 5 [52]. Cooking increases non-acylated anthocyanins, with a higher bioavailability, but not acylated anthocyanins, with a shorter half-life [52]. Non-thermal technologies may reduce ACYs loses from prepared foods [53].

Increasing storage time was associated with degradation of ACYs by many physicochemical factors, explaining why ACYs-based products are not widely used as pigments [53]. ACYs are stable in potatoes, but in pepper and eggplant, concentration decreases upon ripening [13].

Absorption, gastrointestinal transit, and plasma concentration of ACYs depend on their structure, especially on the presence of carbohydrates [52,53]. ACYs are exposed along the gastrointestinal tract, to pH and ions, which affect their bioavailability and bioactivity [53]. An important limitation in most of the studies is lack of control of absorption and metabolism of ACYs [134]. As an example, cranberry anthocyanins are poorly absorbed and rapidly removed from the plasma, with maximal concentrations detectable in plasma between 1 and 3 h, with important differences between study participants [95]. Phytic acid enhances gastrointestinal absorption of ACYs, but is also a strong chelator of minerals, especially iron, and may cause mineral deficiencies, requiring safety tests when used in foods [135]. Most studies do not consider in vivo degradation of ACYs or colonic metabolites [16]. ACYs composition and bioactivity were strongly affected by in vitro gastrointestinal digestion, but antioxidant activity was preserved [136]. Only small amounts of ACYs are excreted in the urine [37].

Bioactive compounds from the same food may act synergistically. Usually, it is impossible to consider all the components of fruits and vegetables: diverse polyphenols, proteins, carbohydrates, fibers, vitamins, and minerals. Food frequency questionnaires might not include all sources of ACY intake. For the moment, there are no biomarkers for ACYs because their metabolites are not well known [16]. It is possible that individuals with a high ACYs intake have a healthier lifestyle, while those with a poor intake have a diet with less fiber, antioxidant vitamins and more saturated fats [38]. Use of extracts from fruits, containing just anthocyanins, might be more effective in assessing their biological actions [37].

A threshold of intake should be defined to obtain cardiovascular benefits. The discrepancies between in vivo and in vitro results may be explained by the high amounts of ACYs, often not physiologically relevant, with low bioavailability in the human body, in most in vitro studies [85]. It is possible that the amount required to achieve a specific biological action is much larger than the one obtained from the diet [53]. Malto/cyclodextrins, liposomes or concentrated sources of ACYs (purees or freeze-dried fruits) are solutions to preserve the bioavailability of ACYs, considering the limited splanchnic metabolism [53]. ACYs are non-toxic molecules within a normal, physiological consumption [53]. No toxicity has been reported for luteolinidin at doses 4-fold above the minimally effective dose, and liposomal delivery enabled rapid cardiac uptake [87].

Dietary choices and the habitual intake of ACYs depend on several factors in different populations, regions, seasons, and individuals with different socio-cultural, ethnic and financial characteristics, as well as technical advances in agricultural and food industry [38,53].

## 5. Future Research Directions

Further large sample size randomized controlled trials need to confirm the effectiveness of anthocyanin supplementation in improving vascular function, structure, and platelet activation. Further in vitro and in vivo studies may identify new chemical and biological aspects of ACYs and provide additional mechanistic knowledge. Further research considering absorption and dose-response effects is warranted. However, solutions must be found to maintain an adequate number of metabolites in plasma and target tissues, considering the nutraceutical effects of ACYs in living systems. The future belongs to foodomic studies, functional food research, phytopharmaceuticals containing ACYs and components with synergistic action, and exploratory epigenetic studies.

A dietary score would be required to compare the anti-inflammatory, antioxidant, anti-atherosclerotic, antihypertensive, antiglycation and antithrombotic properties of ACYs-rich foods, their effect on arterial stiffness, gene expression, phytoestrogenic and ergogenic effects, considering also other components with synergistic action.

## 6. Conclusions

Current positive scientific evidence from epidemiological, observational and intervention studies, randomized controlled trials and mechanistic research, is promising, revealing that anthocyanins represent an inexpensive, accessible, and effective approach, in control of atherosclerosis, cardiovascular risk and cardiovascular aging. The cardiovascular health promoting effects of ACY are possible through multiple mechanisms. Anthocyanins exert favorable effects on the endothelial function, oxidative stress, inhibit COX-1 and COX-2 enzymes, exert antiatherogenic, antihypertensive, antiglycation, antithrombotic and anti-inflammatory activities, ameliorate dyslipidemia and arterial stiffness. Anthocyanins exert also ergogenic effects, probably by influencing vasodilation and relaxation during exercise.

The present review supports the recommendations of the European Society of Cardiology on cardiovascular disease prevention, that cardiovascular risk may be reduced by a diet rich in fruit and vegetables. The role of anthocyanins in the global food chain should increase, and physicians of different specialties and people worldwide should be aware about their health effects in dietary choices. The presented data may help to refine previous dietary recommendations for the slowing of cardiovascular ageing, increasing health- and lifespan and prevention of cardiovascular disorders.

## Figures and Tables

**Table 2:** Key-mechanisms of cardiovascular benefits of anthocyanins (ACYs).

Effects	Mechanisms of Action of ACYs
**Lipid metabolism:** - lower total cholesterol, LDL, non-HDL cholesterol, serum triglycerides, apoprotein B, nonesterified fatty acids	- increase fecal excretion of sterols, down-regulate gene expression of key lipid metabolism enzymes, slow intestinal lipid absorption, increase the expression of LDL-receptors, increase the lipolytic activity of adipocytes [51,53,61]; (100 µM C3G or Cy) [61]
- increase HDL levels [38]	- increase paraoxonase 1 activity (160 mg daily) [68] - increase reverse cholesterol transport mediated by the gut microbiota metabolite, protocatechuic acid (20 nmol/L) [29]
**Carbohydrate metabolism:** - improve glucose tolerance - improve insulin resistance	- impair intestinal absorption of glucose by inhibiting alpha-glucosidase and alpha amylase; protect pancreatic beta-cells from oxidative stress induced by glucose [53,62,63]; (21 mg/kg) [62] - restore IRS-1/PI3K/Akt pathway (C3G >97% purity) [66]
**Anti-obesity**: lower body weight, adipose tissue size, central adiposity, and food intake	- reduce the expression of neuropeptide Y, increase gama-amino butyric acid receptor, reduce protein kinase A-alpha and phosphorylated cAMP-response element binding protein in the hypothalamus [50,64]; (0.037% black soybean - ACYs) [50]; (24 mg/kg for 40 days) [64] - changes adipocytokine expression [62,67,69]; (100 µM C3G or Cy) [61]
**Endothelial function**	- incorporation of ACYs into endothelial cells can protect against insult from oxidative stress [57,70]; (500 g of strawberries for 1 month) [57] - increase the expression of endothelial nitric oxide synthase [71,72]; (purity more than 99.5%) [71] - decrease expression of endothelin-1 (25% delphinidin) [73] - influence the composition of the arterial wall; (54% juice, 835 mg total polyphenols, and 94 mg anthocyanins) [56]
**Vessel wall**	- antioxidant, anti-inflammatory effects, improve endothelial function [57,69,70,74]
**Anti-inflammatory**	- inhibition of nuclear factor kappa beta [75]; (300 mg/day for 3 weeks) [76] - impair expression of adhesion molecules [75]; (Cy3G decreased the adhesion by about 41.8% at 10 μg mL/l, while PrA and GA reduced the adhesion at 1 and at 10 μg mL/l) [77] - impair release of monocyte chemotactic protein (MCP-1); (12 g of an anthocyanin extract) [78] - decrease expression of COX2 in vascular smooth muscle cells (VSMCs) [75]; (anthocyanin mixtures at 66 and 67%, 60 and 72%, and 51 and 76%, respectively) [79] - decrease migration of immune cells (0.24 mg/mL ACYs) [80]
**Antioxidant** (73.53 ± 0.13 mg per 100 g fresh weight basis sample: *Cichorium intybus*) [81]	- quenche singlet oxygen; scavenge hydroxyl and superoxide radicals [37,82] - inhibit prooxidant enzymes and increase activity of antioxidant enzymes [17,57]; (30 mL of cherry concentrate diluted to a volume of 250 mL with water or the same volume) [17] - protect DNA against oxidation [83]
**Anti-atherosclerotic**	- improve endothelial function [48,49]; (purity of ACYs more than 99.5%) [71] - inhibit the proliferation of vascular smooth muscle cells [75]; (the combination treatment of atorvastatin (0.1 µM) and C3G at the concentrations of 2 µM and 20 µM) [84] - reduce the level of oxidized LDL and improve lipid profile [51,53,61,75]; (100 µM C3G or Cy) [61]
**Platelet function**	- reduce the number of activated platelets (500 g of strawberries for 1 month) [57] - lower platelet aggregability [73,85]; (25% delphinidin) [73]
**Antihypertensive**	- inhibit the angiotensin converting enzyme [29,85]; (IC50 = 91.2 μg/mL] [86]

in brackets: the concentration or dose of anthocyanins responsible of the effect observed; CD = cluster of differentiation; C3G = cyanidin 3-glucoside; Cy = cyanidin; Pr = A protocatechuic acid, GA = gallic acid; IC50 = the half maximal inhibitory concentration.

**Table 3:** Vascular effects of anthocyanins. Observational and intervention studies.

Study Population	Anthocyanin Source	Methods	Findings, Conclusions	Ref.
1898 women, 18–75 years old, from the TwinsUK registry	Validated food-frequency questionnaire	PWV, AI, central blood pressure, MAP, IMT	Consumption of 1–2 portions of berries daily reduced arterial stiffness and cardiovascular disease risk	[116]
35 men with mild hypercholesterolemia	Chockeberry juice, 6 weeks regular drinking	NO, FMD, serum lipids	Regular drinking of chockeberry juice improves endothelial function and serum lipids (total and LDL cholesterol and triglycerides) in men with hypercholesterolemia.	[118]
10 patients with carotid atherosclerosis	Pomegranate juice up to 3 years/control group	Common carotid IMT, blood samples	Significant IMT and SBP reduction, serum paraoxonase activity increased, LDL oxidation impaired, decreased antibodies against oxidized LDL, serum antioxidant status increased	[117]

PWV = pulse wave velocity, AI = augmentation index, MAP= mean arterial pressure, IMT = intima-media thickness, NO = nitric oxide, FMD = flow-mediated dilation, SBP = systolic blood pressure, Ref. = references.

**Table 4:** Vascular effects of anthocyanins (ACYs). Randomized controlled trials (RCT).

Study Population	ACY/ Placebo	Methods	Findings, Conclusions	Reference
18 healthy adults	combined ACYs and bromelain supplement (BE)	randomised crossover design; FMD, BP, TAC, resting heart rate, oxygen utility capacity and fatigability measured pre- and post-BE and placebo intake	BE intake is effective for improving endothelial function, BP, TAC and oxygen utility capacity	[125]
14 older adults	7-days 2X 300 mg capsule with 35% *blackcurrant* extract/ placebo	double-blind, placebo-controlled, crossover design study with a washout period of 28 days	ACY intake reduces carotid femoral PWV and central BP in older adults; no effects on blood lipids	[126]
19 patients, 20 to 60 years old, with metabolic syndrome (MetS)	240 mL of tart *cherry juice* (rich in ACYs) or an isocaloric placebo-control drink, twice daily for 12 weeks	single-blind, placebo-controlled, parallel-arm pilot clinical trial PWV, brachial and aortic BP, AI, and biomarkers of cardiovascular and metabolic health, assessed at baseline and 6 and 12 weeks	no significant changes in hemodynamics and arterial stiffness lower oxidized low-density lipoprotein, soluble vascular cell adhesion molecule-1 and total cholesterol after tart cherry juice than control	[127]
15 healthy overweight and obese men and women	200 mL blood orange juice twice daily) for 2 weeks with a washout period of 1 week	primary outcome: FMD	favorable effects on endothelial function	[120]
115 participants, age 63 ± 7 years; 68% male	daily intake of 1 cup (150 g) of *blueberries* for 6 months	double-blind, parallel RCT; insulin resistance, FMD, AI, lipoprotein status, and NO	improvements in vascular function, lipid status, and NO bioactivity	[121]
41 participants, aged 25–84 years	500 mL blood *orange juice* providing 50 mg ACYs/ 500 mL blonde orange juice without ACYs for 28 days	open label, two-arm cross-over trial; total, HDL- and LDL-cholesterol, glucose, fructosamine, NO, CRP, aortic SBP and DBP or carotid-femoral and brachial-ankle PWV	No significant differences were observed between the variables measured at the start and end of each treatment period. The lack of effect may be due to the modest concentration of ACYs in the blood orange juice	[128]
14 healthy male and female adults	Participants consumed 200 g/day of cooked *purple potato* containing 288 mg ACYs, or a white potato containing negligible ACYs for 14 days, separated by a 7-day washout period.	PWV, SBP, DBP, HDL, LDL, TG, glucose, insulin, and CRP.	PWV was significantly reduced following purple potato consumption for 14-days	[22]
60 postmenopausal women with pre- and stage 1-hypertension	8 weeks, 25 g or 50 g freeze-dried *strawberry powder* (FDSP)	double-blind, placebo-controlled, parallel arm clinical trial BP, arterial stiffness, superoxide dismutase (SOD) at baseline, 4 and 8 weeks	BP and arterial stiffness improved in the 25 g FDSP group	[129]
13 healthy men, age: 25 ± 4 years	New Zealand blackcurrant (NZBC) extract (600 mg/day)/ placebo for 7-days separated by 14-days washout	double-blind, crossover design, Participants produced isometric maximal voluntary contractions (iMVC) and a 120-s 30%iMVC of the quadriceps: electromyography, near-infrared spectroscopy, hemodynamic and ultrasound recordings	Intake of NZBC extract impaired cardiovascular responses, muscle oxygen saturation, muscle activity and femoral artery diameter of the quadriceps and may increase exercise performance	[21]
16 volunteers performing a single standard dive	2 groups: one of them received 2x 200 mg of an ACYs-rich extract from red oranges, 12 and 4 h before diving	FMD	ACYs administration reduces the harmful endothelial effects of a recreational single dive	[124]
48 postmenopausal women with pre- and stage 1 hypertension	8-week, 22 g freeze-dried blueberry (BB) powder/control daily	double-blind, placebo-controlled clinical trial BP, PWV, CRP, NO and SOD at baseline, 4 and 8 weeks	Daily BB reduces BP and arterial stiffness, related to increase of NO production	[90]
25 men and postmenopausal women, 18–50 years old	6 weeks, 250 g BB powder/ placebo daily	BP, vascular performance testing, blood samples at baseline and after 6 weeks	BB ingestion for 6 weeks increases natural killer cells and reduces AI, SBP, DBP in sedentary males and females	[111]
47 healthy adults, 30–50 years	6 weeks, 30 mL tart cherry juice concentrate diluted with water/energy matched control drink	BP, arterial stiffness, CRP, total cholesterol, LDL, ferric reducing ability of plasma at baseline and after 6 weeks	Tart cherry juice concentrate has no effect on arterial stiffness, CRP, and cardiovascular risk markers, but increases antioxidant status	[17]
21 healthy men	766, 1278 and 1791 mg blueberry polyphenols (BBPP)/Control 319, 637, 766, 1278, 1791 mg total blueberry/ control	Double-blind, controlled, crossover trial; FMD Intake-dependence study, from baseline to 1 h	FMD increased significantly at 1–2 and 6 h after consumption of BBPP. At 1 h after consumption, FMD increased dose-dependently to up to 766 mg BBPP. The vascular benefits are linked to the circulating phenolic metabolites and activity of the neutrophil NADPH oxidase	[130]
11 young, healthy male nonsmokers and 13 smokers	supplement A (50 mg of blackcurrant ACY) and supplement B (50 mg of blackcurrant anthocyanin plus vitamin E	Double-blind trial; FMD and skin temperature	Oral ACYs and Vitamin E supplementation can attenuate the smoking-induced acute endothelial dysfunction and peripheral blood flow in smokers	[123]
44 patients with coronary artery disease	480 mL of *cranberry juice*/placebo for 4 weeks	BP, PWV, brachial artery flow-mediated dilation, digital pulse amplitude	Chronic cranberry juice consumption reduced arterial stiffness, with only an acute benefit on endothelial vasodilator function	[56]
12 patients with hypercholesterolemia 150 hypercholesterol-emic individuals	320 mg ACYs/ placebo 320 mg ACYs/ placebo	FMD before and after the intervention FMD, cGMP	ACYs supplementation improves endothelium-dependent vasodilation in patients with hypercholesterolemia, related to activation of the NO-cGMP signaling pathway, improvement of serum lipids and an anti-inflammatory effect	[72]
Subjects at moderate risk for coronary heart disease	240 mL *pomegranate juice*/day (n- = 146)/control bevarage (n = 143) up to 18 months	IMT	No significant effect of pomegranate juice was noticed on IMT progression rate. A slowed IMT progression was noticed in patients with increased oxidative stress and impaired TG/HDL profile	[131]
9 healthy men	17 mg kg(-1) BCA or placebo	double-blind, placebo-controlled, crossover study NIRS, improvement in shoulder stiffness plasma ACYs measured prior to ingestion and 1, 2, and 4 h later	FBF increased significantly after BCA ingestion	[119]

PWV = pulse wave velocity, FMD = flow-mediated dilation, AI = augmentation index, NO = nitric oxide, BP = blood pressure; SBP = systolic blood pressure, DBP = diastolic blood pressure, HDL = high-density lipoproteins, LDL = low-density lipoproteins, TG = triglycerides, CRP = C-reactive protein; FDSP = freeze-dried strawberry powder; iMVC = isometric maximal voluntary contractions; NZBC = New Zealand blackcurrant extract; BB = freeze-dried blueberry; SOD = superoxide dismutase; BBPP = blueberry polyphenols; BCA = blackcurrant anthocyanin; NIRS = near-infrared spectroscopy; FBF = left forearm blood flow; TAC = total antioxidant capacity.
